# Effects of Temperature on the Morphology and Optical Properties of Spark Discharge Germanium Nanoparticles

**DOI:** 10.3390/ma13194431

**Published:** 2020-10-05

**Authors:** Anna Lizunova, Anastasia Mazharenko, Bulat Masnaviev, Egor Khramov, Alexey Efimov, Andrei Ramanenka, Ivan Shuklov, Viktor Ivanov

**Affiliations:** 1 Moscow Institute of Physics and Technology, National Research University, 141700 Moscow, Russia; mazharenko.a@phystech.edu (A.M.); masnaviev.bi@phystech.edu (B.M.); egor.khramov@phystech.edu (E.K.); efimov.aa@mipt.ru (A.E.); shuklov.ia@mipt.ru (I.S.); ivanov.vv@mipt.ru (V.I.); 2 Stepanov Institute of Physics of the National Academy of Sciences of Belarus, 220072 Minsk, Belarus; a.ramanenka@ifanbel.bas-net.by

**Keywords:** germanium nanoparticles, spark discharge, photoluminescence, Mie theory

## Abstract

We report the spark discharge synthesis of aerosol germanium nanoparticles followed by sintering in a tube furnace at different temperatures varying from 25 to 800 °C. The size, structure, chemical composition and optical properties were studied. We have demonstrated a melting mechanism of nanoparticles agglomerates, the growth of the mean primary particle size from 7 to 51 nm and the reduction of the size of agglomerates with a temperature increase. According to transmission electron microscopy (TEM) and Fourier transform infrared (FTIR) data, primary nanoparticles sintered at temperatures from 25 to 475 °C basically have a structure of Ge crystals embedded in a GeOx amorphous matrix, as well as visible photoluminescence (PL) with the maximum at 550 nm. Pure germanium nanoparticles are prepared at temperatures above 625 °C and distinguished by their absence of visible PL. The shape of the experimental UV-vis-NIR extinction spectra significantly depends on the size distribution of the germanium crystals. This fact was confirmed by simulations according to Mie theory for obtained ensembles of germanium nanoparticles.

## 1. Introduction

Semiconductor nanoparticles (NP), in particular silicon and germanium, continue to be a subject of interest for achieving enhanced properties of light emitters in various quantum technologies, especially for optoelectronic devices and biomedical applications [1,2,3,4].

The distinguished advantages of germanium in comparison to silicon are its higher exciton Bohr radius, so the quantum confinement effects can be observed at a larger size (~24 nm), and a narrow band gap at 300 K (0.67 eV), which suggests the possibility of emission over a wide range, from ultraviolet (UV) through the visible to the near-infrared (NIR, ~1.7 mkm) [3].

Since germanium-based photoluminescent nanostructures can be easily integrated into silicon chips, they are used as light sources in optoelectronic devices. Metal-oxide-semiconductor (MOS) memory devices reduced leakage current and writing voltage in case of embedding germanium nanoparticles in oxide layers in the floating gate [5]. Germanium nanoparticles are a promising material for replacement of carbon in lithium battery anodes and allow for 10 times increase in a volume capacity [6].

Unique photoluminescent properties were discovered for germanium nanoparticles both for colloids [7] and MOS structures, based on embedding germanium nanoparticles in silicon structures [8,9]. It is known that by varying the size of nanoparticles from 2 to 5 nm it is possible to alter the photoluminescence (PL) of the germanium colloid particles over a wide range from 300 to 1600 nm [10,11]. The greatest effect of the influence of size on the PL properties occurs when the nanoparticle size varies from 1 to 5 nm. A few researches studied the optical properties of larger size Ge NP [12] and no strong confinement effects were observed in the visible PL and the absorption of germanium NP with sizes from 8 to 25 nm [13,14]. A sufficient number of publications reveal germanium nanoparticles and core-shell structured Ge/GeOx luminescence belonging to the visible spectrum range [15,16]. Nevertheless, the maxima of luminescence peaks are affected not only by the size of the particles, but also by the size distribution width, structure, method of synthesis and surface properties [7,8,9,10,11,12,13,14,15,16,17]. Thus, a definition of the correlation between optical size and structural properties of germanium nanoparticles is still a challenging task.

It is known that the synthesis of NPs by spark discharge [18] is the most promising method to produce nanoparticles for aerosol jet printing technology [19], with the goal to form microstructures for biomedical and optoelectronic applications. Since the spark discharge method allows control of the size, crystal structure, elemental composition and other physical properties of NPs, depending on synthesis conditions [20,21], it is a powerful technique for studying the photoluminescent characteristics of various materials, including nanoparticles of metals and semiconductors [18,19,20,21,22,23,24]. Therefore, a thorough knowledge of the optical properties of germanium nanoparticles produced by different methods is required to progress in microelectronics, biosensors development and further evolution of IR devices.

The goal of the present study is an investigation of the special aspects of germanium nanoparticle production by the spark discharge method in ultrapure argon atmosphere, with additional in-flow sintering at temperatures ranging from 25 to 800 °C. In a previous paper we showed that the sintering temperature can significantly alter the morphology and the size of silver nanoparticles [22]. Taking into account the sensitivity of optical properties towards size variations, the main task of the project was to obtain germanium nanoparticles with a wide range of dimensions and to investigate the evolution in their structure, morphology, photoluminescence and extinction properties caused by high temperatures, as well as to reveal the dependency of optical properties on size and structure characteristics. Furthermore, Mie theory average extinction cross-sections were calculated for the obtained particle distributions, in order to confirm size-dependent absorption properties.

## 2. Materials and Methods

**Figure 1:**
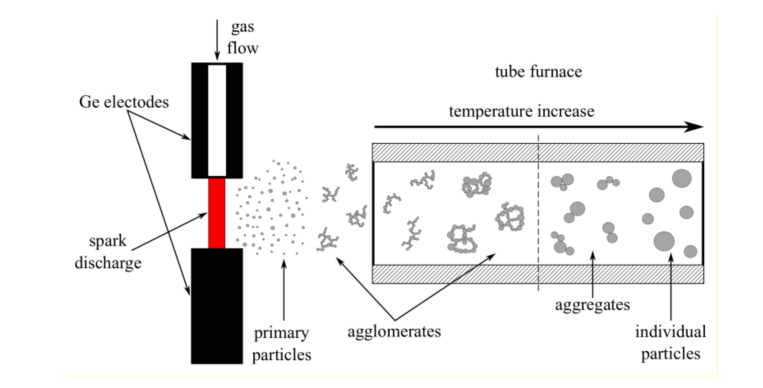
The schematic of the formation of nanoparticles in the spark-discharge aerosol generator, supplemented with the tube furnace heated to different temperatures. The temperature of the tube is constant along the whole length of the furnace. One can affect the morphology and size of nanoparticles by controlling the temperature.

**Figure 2:**
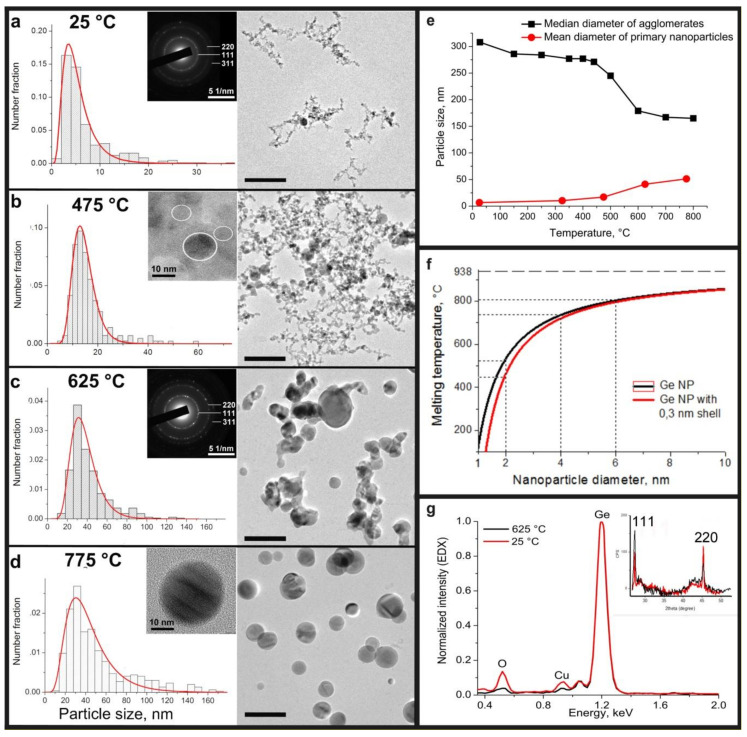
Evolution in morphology and particle size of germanium nanoparticles with increasing temperature: transmission electron microscopy (TEM) images and corresponding particle size distributions for samples, sintering at (**a**) 25, (**b**) 475, (**c**) 625 and (**d**) 775 °C. The scale bar is 100 nm. Typical HRTEM images: insert (**b**) Ge crystals in a GeOx amorphous matrix, sintered at 25–475 °C; insert (**d**): pure Ge crystal nanoparticles, obtained at high temperature synthesis (above 625 °C); inserts (**a**), (**c**): a typical SAED pattern of prepared germanium nanoparticles with temperature sintering from 25 to 775 °C; (**f**): the dependence of the melting temperature on the diameter of the germanium nanoparticles; (**g**): typical normalized EDX spectra of nanoparticles sintered at low (25–475 °C) and high (above 625 °C) temperatures and corresponding XRD spectra obtained from a Ge NP on cellulose filter (insert).

Germanium nanoparticles were produced using the aerosol spark discharge generator developed in our laboratory [20]. The system was supplemented by a tube furnace for nanoparticle sintering at different temperatures in gas flow (Figure 1) [22]. Electrode material is evaporated in the environs of the spark, initiated by gas breakdown under high-power current impulses. The erosion produces atomic clusters, which agglomerate and form primary nanoparticles with a size of 5 to 8 nm [19,20,21,22,23]. As the primary nanoparticles move further along the gas path, they collide and stick together, forming fractal-like agglomerates (Figure 2a). When the produced agglomerates move through the heated furnace with constant temperature along the entire length of the tube, partial or complete melting of the particles occur, depending on the furnace temperature, with a subsequent process of recrystallization into new-made particles.

Cylindrical n-type germanium, whose specific resistance was 0.005 Ohm∙cm, was used as the material for electrodes for electrical erosion [23]. Firstly, the chamber volume was pumped to a pressure of 12 Pa. Spark processing was performed in argon gas of 99.9999 purity, which was fed into the synthesis chamber through one of the electrodes (anode). The pressure of the argon gas in the camera setup was maintained at approximately 1.5 bar. The frequency of the discharges and the capacitor voltage were 370 Hz and 1.5 kV, respectively.

Five samples of germanium nanoparticles were prepared at different sintering temperatures of the tube furnace: 25, 325, 475, 625 and 775 °C. Aerosol nanoparticles were collected on a cellulose filter and transmission electron microscopy (TEM) copper grids with carbon film directly in the setup chamber.

The particle size, distribution, morphology and crystal structure of the samples were analyzed with the aid of transmission electron microscopy (TEM) Jeol JEM 2100 (200 kV) with energy dispersive X-ray spectrometer X-MAXN OXFORD instruments. The agglomerates size distribution in the flow was measured using a TSI SMPS 3936 aerosol spectrometer. Fourier transform infrared (FTIR) spectra in attenuated total reflection mode were recorded on a Nicolet™ iS50 FTIR spectrometer (Thermo Scientific) directly from the germanium nanoparticles on cellulose filter. The UV-vis-NIR spectra of nanoparticles dispersions in a quartz cuvette were obtained using a JASCO V–770 spectrophotometer. Photoluminescence spectra of the solutions of germanium nanoparticles in the visible range were collected using a Spectrofluorometer JASCO FP-8300 with a continuous xenon source (150 W) at an excitation wavelength of 300 nm. To investigate the obtained nanoparticles with a spectrophotometer and a spectrofluorometer, the nanoparticles were precipitated from the cellulose filter and diluted with isopropyl alcohol (LiChrosolv, Merck), then sonicated for 3 min. PL spectra in the NIR region (800–1600 nm) were registered at room temperature using a Fluorolog-3 spectrofluorometer (HORIBA Scientific, USA-France-Japan). The excitation source was a standard continuous Xe lamp of the spectrofluorometer (450 W); the detector was a Symphony II InGaAs CCD array (HORIBA Scientific) cooled by liquid nitrogen (−103 °C). The emission monochromator used in the experiments was an iHR 320 (HORIBA Scientific). Aerosol Ge nanoparticles collected on a cellulose filter were analyzed by X-Ray diffraction analysis (XRD) using a Thermo Scientific ARL X’TRA X-Ray diffractometer equipped with a parabolic mirror (AXO Dresden) and a pinhole collimator.

## 3. Results and Discussion

### 3.1. Size Analysis

TEM images of Ge nanoparticles sintered at different temperatures are presented on Figure 2; they show different morphologies of the particle ensembles. Nanoparticles prepared at room temperature form large fractal-like structure agglomerates of primary particles, which are mostly rather spherical and measure 2–15 nm in diameter. Very few spherical crystal Ge particles that are much larger, up to 37 nm, are detected. The material represents the mean diameter of primary particles 6.8 ± 5.2 nm and follows a lognormal particle distribution (Figure 2a), typical for spark discharge processes [19,20,21,22]. The same particle morphology was observed for the samples, prepared at 325 and 475 °C, with the mean primary particle sizes 10.4 ± 7.2 and 17.0 ± 10.3 nm, respectively. However, with a further increase in temperature up to 625 °C, the particle size increased by more than two times to 41.2 ± 19.1 nm. Thus, according to microscopic image analysis, a sintering temperature increase causes the mean particle size for a sample to grow from 6.8 ± 5.2 at 25 °C to 51.3 ± 31.8 nm at 775 °C. The increase of distribution width can also be observed in Figure 2. In comparison with the 25 °C sample, the range of obtained particle sizes varies from 2 to 35 nm, whereas for germanium, produced at 775 °C, the size of particles ranged within 14 to 170 nm.

The sintering temperature also strongly affects the particle morphology. Agglomerates of nanoparticles appeared to be Ge crystals embedded in the GeOx matrix were grown at low temperatures, from 25 to 475 °C. A typical high resolution TEM image of nanoparticles with a germanium core and probably a germanium oxide amorphous shell (thickness of 1–5 nm) joined by an amorphous neck, obtained at temperatures from 25 to 475 °C, is shown on the insert of Figure 2b. Otherwise, most of the particles prepared at 625 and 775 °C are pure germanium crystals without an amorphous matrix (Figure 2d). According to the TEM images (Figure 2c,d), strongly coupled aggregates of Ge crystals in the sample produced at 625 °C were observed, whereas during sintering at 775 °C particles were mostly spherical and freestanding. 

The selected area electron diffraction (SAED) patterns of germanium particles sintered at different temperatures have identical rings with different intensity and contain only a diamond-like lattice structure of germanium (insert on Figure 2a,c). This data confirms our previous XRD experiments for room temperature synthesis of Ge nanoparticles, presented in the study [24], only narrow peaks from cubic germanium phase are observed (insert on Figure 2g). EDX analysis shows (Figure 2g) the presence of oxygen in the germanium agglomerates prepared below 475 °C. There is only a residual amount of oxygen in the microscope chamber for the high-temperature samples (above 625 °C). The detected peak of copper is a usual artifact of the TEM analysis and it appeared from the copper TEM grid. The absence of additional peaks on XRD patterns justifies the presence of strictly amorphous germanium oxide.

A change in the median diameter of the agglomerates with a sintering temperature, measured in argon flow by the aerosol spectrometer, shows only a small decrease in size in the range from 400 to 700 °C from ~280 to ~170 nm (Figure 2e). While the mean size of the primary nanoparticles obtained by TEM goes up with the increase in temperature, the median diameter for agglomerates is in decline. TEM images show the particle morphology change, which is caused by a process of progressive melting of the primary nanoparticles in agglomerates, resulting in the formation of big liquid drops and recrystallization.

It is known that the melting temperature depends on the particle size and thermodynamic parameters of the material [25] as described in the following equation

(1) $$T_{\mathrm{NP}}=T_{\mathrm{bulk}}\left(1-\frac{4\sigma }{{\Delta H}_{m}\rho_{S}d}\right),$$

where $T_{\mathrm{NP}}$ is the melting temperature of nanoparticles; $T_{\mathrm{bulk}}=938\;{}^{\circ}C$, the melting temperature of bulk germanium; σ=0.583 N/m, the surface tension at the liquid-solid interface; $\rho_{S}=5320\;\mathrm{kg}/m^{2}$, the density of solid germanium; ${\Delta H}_{m}=508,800\;J/g$, the enthalpy of fusion for germanium; and d is the diameter of nanoparticles.

It means, that the melting point of the particle is strictly defined by its size, significantly while the diameters of the particles are in the range of 1 to 10 nm (Figure 2f). At temperatures around 450 °C particles sized 2 nm in diameter start to melt and become an impulse for the larger particles melting. The presence of a liquid film diminishes the melting point for those larger particles at the rate of $1/\left(r-l\right)$ [26], where *l* is the thickness of the liquid shell. As can be observed from the histograms on Figure 2a,d, the fraction of the smaller particles goes down as temperature is increased. The minimal size of particles grows with an increase of temperature, since the smaller particles melt and become a shell for the larger ones. The presence of the liquid shell initiates melting of the larger particles at temperatures lower than calculated by Equation (1). As a result, all agglomerates produced at spark discharge transshape to monolithic spherical germanium particles during the sintering in the furnace at 775 °C, whereas a temperature near 800 °C is estimated to be the melting point for particles 6 nm in diameter. This size is close to the mean diameter of primary nanoparticles produced in spark discharge at room temperature (25 °C), without any additional heat treatment. Notably, the melting process of nanoparticle agglomerates takes place at temperatures much lower than the temperature calculated for an agglomerate of initial size (320 nm). The estimated melting point for 320 nm particles using the Equation (1) is equal to 936 °C and it is close to the melting temperature of bulk germanium. Thus, the melting of the agglomerates is strongly defined by the size of the primary nanoparticles, which formed the agglomerate, not the size of the whole agglomerate.

### 3.2. FTIR Measurements

**Figure 3:**
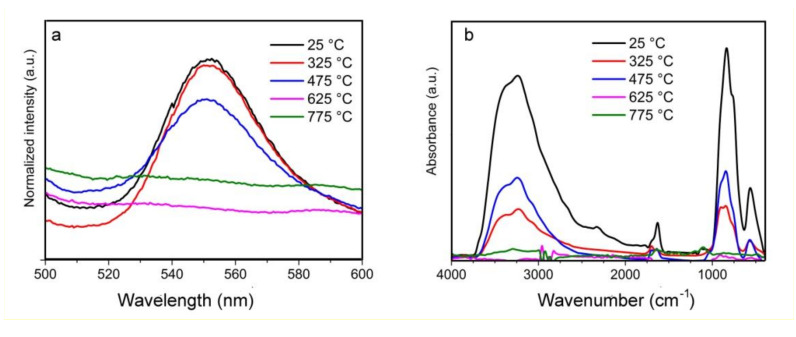
(**a**) Photoluminescence (PL) luminescence and (**b)** Fourier transform infrared (FTIR) spectra of germanium nanoparticles prepared at different temperatures.

FTIR absorption spectra of germanium nanoparticles synthesized at 5 different temperatures are shown on a Figure 3b. To obtain the presented spectra the filter spectrum was subtracted from the initial spectra of germanium nanoparticles on the filter. Based on this figure, the main absorption bands are at ~566, ~820, ~1638, ~1702 and a broad peak from 3000 to 3600 cm^−1^ could be clearly observed for the samples, produced at low temperatures from 25 to 475 °C. None of the peaks were detected for samples produced at high sintering temperatures (625 and 775 °C).

Two intense Ge-O bands are observed at 566 and 820 cm^−1^. They are attributed to the Ge-O-Ge bending vibration mode and to the stretching vibration mode of the same group, respectively [27]. It exhibits a strong O-H stretching peak at ~3400 cm^−1^, which is assigned to the overlapping O-H stretching mode of water (~3100 cm^−1^) and the surface Ge-OH band (~3670 cm^−1^). A much weaker band around 1644 cm^−1^ is also attributed to be from H-O-H bending.

According to the FTIR spectra, the absorbance for IR waves in the range of 3750 to 2500 cm^−1^ and 1000 to 400 cm^−1^ drops drastically with an increase of sintering temperature. This can be explained by the reduction of number of bonds absorbing in those ranges. This fact is caused by extreme heating of the nanoparticles resulting in deoxidization of germanium oxide in inert atmosphere to pure semiconductor. As a result, it can be observed that the intensities of the GeO peaks decrease with an increased temperature from 25 to 475 °C, and the peaks disappear for germanium nanoparticles, produced at the temperatures 625 and 775 °C.

### 3.3. Photoluminescence Analysis

The PL spectra shown in Figure 3a exhibit a photoluminescence peak in the yellow–green wavelength range with a maximum at 550 nm only for particles synthesized at low temperatures (25–475 °C) and characterized by a germanium core and amorphous shell of germanium oxide. The maximum of the PL peak does not shift and has a width from 520 to 580 nm, the same as for the three samples. When the temperature treatment is above 625 °C, the germanium oxide is reduced to pure semiconductor germanium in an inert atmosphere of argon, and the presence of a Ge-GeOx crystal-matrix structure is not detected, and a visible PL emission is not detected. Thus, we suggest that luminescence is caused by surface states at the Ge/GeOx interface or by recombination in a thin germanium oxide layer. Similar conclusions were reached by a number of researchers working both with (1) germanium nanoparticles embedded in germanium oxide films [16,17,18,19,20,21,22,23,24,25,26,27,28], observing luminescence in the range of 400 to 660 nm, and (2) studying colloidal synthesis of Ge/GeO_2_ core-shell germanium nanoparticles; in that case they detected a weak luminescence peak at 500 nm [15]. The presence of oxygen in the samples with small particle size might be due to rapid oxidation of the particles’ surfaces during storage and while conducting experiments in air atmosphere.

To prove our suggestion about the nature of obtained photoluminescence, we studied near-infrared PL of Ge nanoparticles. According to the work [29] the intensity of NIR luminescence from quantum-confined excitons in Ge NP decrease by an order of two while the size of the particles increases from 1 to 5 nm. Any PL peaks were not detected in the range from 800 to 1600 nm. This is probably due to the low number of nanoparticles with sizes below 3 nm, which significantly contributes to NIR PL.

According to the facts above we conclude that obtained luminescence is caused by defect centers in surface states at the Ge/GeOx interface, and no size effects of germanium particles on the position of the luminescence peak were observed. No strong quantum confinement effects were investigated for 8–250 nm germanium nanoparticles [12,13].

### 3.4. Absorption Measurements and Simulations

**Figure 4:**
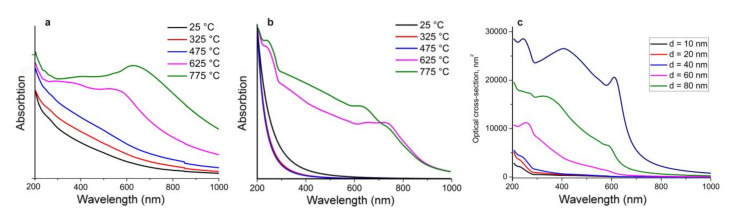
(**a**) Experimental and (**b**) simulated absorption spectra of germanium nanoparticles, sintered at different temperatures, and (**c**) calculated extinction optical cross-section of germanium nanoparticles of different diameters.

Figure 4a shows the typical optical absorbance for five samples of germanium nanoparticles. The absorbance of germanium particles, produced at low temperatures from 25 to 475 °C with mean particle sizes from 5.8 to 17.0 nm characterized by a uniformly decreasing function of wavelength in a wide range from 200 to 1000 nm. In contrast, the absorbance spectra of nanoparticles, sintered at temperatures of 625 and 775 °C, contain some dissimilarities, namely, a maximum at 243 nm, the bend of a curve around 300 nm, almost a plateau over the 300 to 520 nm range, slight peaks at 560 and 630 nm for nanoparticles ensembles with 41.4 and 51.3 nm mean size, respectively. Further continuous decrease of extinction from the red visible to the IR region is observed.

To provide deep insight into the nature of the shape transformation of the absorbance curves for germanium nanoparticles, synthesized at different temperatures, rigorous Mie theory simulations were accomplished [30]. According to electron microscopy analysis (Figure 1) none of the obtained samples is monodisperse; all five samples contain particles in a rather wide rage. In particular, germanium particles with sizes from 2 to 37 nm exist in the sample prepared at 25 °C, while particles with 6 to 60 nm diameters occur in the material produced at 475 °C, whereas particles with diameters from 14 to 170 nm were found in the sample sintered at 775 °C. We suggest that the discrepancy in optical absorbance appears to be due to a different size distribution of the germanium nanoparticles in different samples, as it has been shown for plasmon resonance peak shape transformation for polydisperse aluminum nanoparticles [31]. According to the Mie theory, the extinction cross-section could be presented as a sum of multipole harmonic series [32,33]. Taking into account data presented in the work [34] on Ge complex refractive index spectrum, we calculated the average extinction cross-section of nanoparticles in correspondence with the obtained distribution. For the particles with an amorphous matrix we take into consideration the presence of an amorphous shell with a refractive index of 1.7 for GeO_2_.

Calculated extinction spectra for germanium nanoparticles ensembles are presented on Figure 4b. In the calculated spectra of monodisperse particles (Figure 4c), with an increase in the particle size above 60 nm, higher-order modes (quadrupole, octupole, etc.) appear along with the dipole mode of electron oscillations. Since the absorption cross section for large particles is higher than that of the fine fraction, peaks characteristic of large particles appear in the total spectrum for the ensemble of polydisperse particles. For simulation data of the ensembles, similar changes in the extinction spectra are observed as compared with those of the experimental ones.

For particles with a mean size under 17.0 nm, the ordinary decreasing absorption functions of wavelength as for small monodisperse germanium particles were exhibited. For particles including a small amount of particles with sizes over ~60 nm (germanium nanoparticles ensembles produced at 625 and 775 °C and mean sizes of 41.4 and 51.3 nm, respectively), as well as a wide distribution by size, absorbance peaks at 245 nm and an inflection around 290 nm with a change of an incline and slight maximums in the range of 610 to 740 nm were observed in simulated extinction spectra. Experimentally measured and calculated absorbance peaks in the visible red spectral region show minor discrepancies. An initial reason for this is that cross-particle interactions are not taken into account. As it was mentioned, in Mie simulation we use theoretical optical data for germanium and germanium oxide drawn from works of other authors. Thus, another factor resides in the possible difference between the real characteristics of our samples and the theoretical data on which we relied.

## 4. Conclusions

In summary, germanium nanoparticles of various morphologies were obtained in a wide range of sizes from 2 to 170 nm using a spark gas discharge method with additional heating of particles in an argon flow at temperatures from 25 to 775 °C. The process of agglomerate melting was described. It was found that the melting point of agglomerates is strongly defined by the size of the primary nanoparticles, which formed the agglomerate, rather than the size of the whole agglomerate. The agglomerates of germanium crystals in amorphous oxide matrix with average sizes from 5.8 to 17.0 nm were produced at temperatures less than 475 °C. Visible photoluminescence with a constant maximum position at 550 nm was detected for Ge/GeOx particles, and no PL quantum confinement effects were established. The absorption of such germanium nanoparticles is characterized by a smooth, decreasing function of a wavelength. In contrast, at temperatures above 625 °C, the effect of disproportionation of germanium oxide to pure germanium was observed, so particles with the morphology of Ge crystals embedded into the oxide matrix transforms into pure semiconductor particles without any luminescence in the visible spectrum. The ensembles of particles, prepared at 625 and 775 °C, were distinguished by larger mean sizes up to 51.3 nm, wider particle size distributions and non-typical absorption spectrum with a sharp bend of about 300 nm and slight peaks in the range of 570 to 630 nm. Mie theory simulations for ensembles of nanoparticles with obtained particle size distribution confirmed that the changes in the absorption function of germanium nanoparticles were related to the size of the nanoparticles. It is shown that the presence of large germanium particles with diameters greater than ~60 nm caused the existence of absorption maxima in the wavelength range from 570 to 750 nm, and the inflection of the absorption function in the UV range.

Thus, production of germanium nanoparticles with different morphologies, sizes and optical properties by the spark discharge method can be controlled by the temperature of an additional sintering process. This is quite important technology for creating nanoparticles with required properties for applications as anode materials of lithium-ion batteries, sodium-ion batteries [35] and aerosol jet-printing devices [36,37,38].
